# The orbitofrontal cortex, food reward, body weight and obesity

**DOI:** 10.1093/scan/nsab044

**Published:** 2021-04-08

**Authors:** Edmund T Rolls

**Affiliations:** Oxford Centre for Computational Neuroscience, Oxford, UK; Department of Computer Science, University of Warwick, Coventry, UK

**Keywords:** taste, olfaction, food reward, food

## Abstract

In primates including humans, the orbitofrontal cortex is the key brain region representing the reward value and subjective pleasantness of the sight, smell, taste and texture of food. At stages of processing before this, in the insular taste cortex and inferior temporal visual cortex, the identity of the food is represented, but not its affective value. In rodents, the whole organisation of reward systems appears to be different, with reward value reflected earlier in processing systems. In primates and humans, the amygdala is overshadowed by the great development of the orbitofrontal cortex. Social and cognitive factors exert a top-down influence on the orbitofrontal cortex, to modulate the reward value of food that is represented in the orbitofrontal cortex. Recent evidence shows that even in the resting state, with no food present as a stimulus, the liking for food, and probably as a consequence of that body mass index, is correlated with the functional connectivity of the orbitofrontal cortex and ventromedial prefrontal cortex. This suggests that individual differences in these orbitofrontal cortex reward systems contribute to individual differences in food pleasantness and obesity. Implications of how these reward systems in the brain operate for understanding, preventing and treating obesity are described.

## Introduction

Research is described at the neuronal level that shows that in primates, the reward value of the sight, smell, taste and oral texture of food is represented in the orbitofrontal cortex, but not at earlier stages of processing. It is shown that this is a different type of organisation from what appears to be present in rodents. Research is then described, which shows in human (fMRI) Functional Magnetic Resonance imaging investigations that the organisation is similar to that in other primates, in that the pleasantness of the sight, smell, taste and oral texture of food is represented in the orbitofrontal cortex, but not at earlier stages such as in the taste insula. This is extended, by showing that in humans, social and cognitive factors such as word-level information that the food is rich and delicious modulates the activations produced by the smell and taste of food in the orbitofrontal cortex. Moreover, paying attention to the pleasantness of the food rather than its physical properties increases activations produced by food reward in the orbitofrontal cortex. Then, it is shown that even in the resting state, when no food is present, the liking of the individual for sweet foods, and as a probable consequence, the body mass index (BMI), is related to the functional connectivity of the reward-related orbitofrontal cortex with action-related systems such as the anterior cingulate cortex. This is related to individual differences in food reward systems that arise, it is proposed, by variation useful in evolutionary processes. This provides a foundation for understanding food reward systems in the brain, and their relation to appetite control and body weight, in humans.

**Fig. 1.**
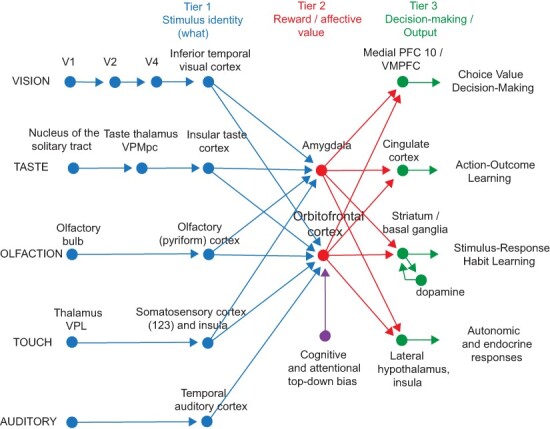
Schematic diagram showing some of the gustatory, olfactory, visual and somatosensory pathways to the orbitofrontal cortex, and some of the outputs of the orbitofrontal cortex, in primates. The secondary taste cortex and the secondary olfactory cortex are within the orbitofrontal cortex. V1—primary visual cortex. V4—visual cortical area V4. Tier 1: the column of brain regions including and below the inferior temporal visual cortex represents brain regions in which ‘what’ stimulus is present is made explicit in the neuronal representation, but not its reward or affective value, which are represented in the next tier of brain regions (Tier 2), the orbitofrontal cortex and amygdala, and in the anterior cingulate cortex. In Tier 3 areas beyond these such as medial prefrontal cortex area 10, choices or decisions about reward value are taken (Rolls, 2008b, 2014; Rolls and Deco, 2010). Top-down control of affective reward systems by cognition and by selective attention from the dorsolateral prefrontal cortex is also indicated. Medial PFC 10/VMPFC—ventromedial prefrontal cortex area 10; VPMpc—ventralposteromedial thalamic nucleus, the thalamic nucleus for taste.

**Fig. 2.**
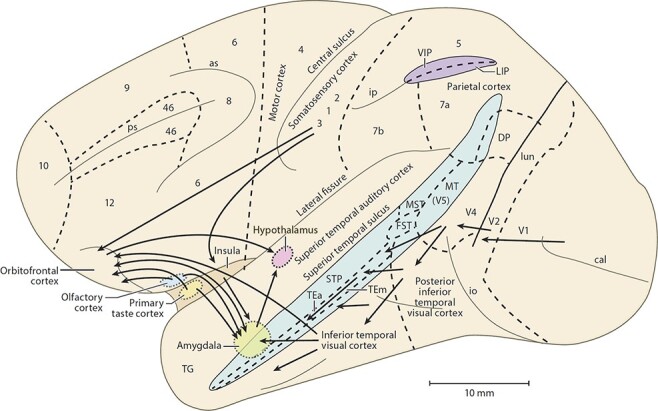
Some of the pathways involved in processing food-related stimuli are shown on this lateral view of the primate brain (macaque). Connections from the primary taste and olfactory cortices to the orbitofrontal cortex and amygdala are shown. Connections are also shown in the ‘ventral visual system’ from V1 to V2, V4, the inferior temporal visual cortex, etc., with some connections reaching the amygdala and orbitofrontal cortex. In addition, connections from the somatosensory cortical areas 1, 2 and 3 that reach the orbitofrontal cortex directly and via the insular cortex and that reach the amygdala via the insular cortex are shown. as, arcuate sulcus; cal, calcarine sulcus; cs, central sulcus; lf, lateral (or Sylvian) fissure; lun, lunate sulcus; ps, principal sulcus; io, inferior occipital sulcus; ip, intraparietal sulcus (which has been opened to reveal some of the areas it contains); sts, superior temporal sulcus (which has been opened to reveal some of the areas it contains). AIT, anterior inferior temporal cortex; FST, visual motion processing area; LIP, lateral intraparietal area; MST, visual motion processing area; MT, visual motion processing area (also called V5); PIT, posterior inferior temporal cortex; STP, superior temporal plane; TA, architectonic area including auditory association cortex; TE, architectonic area including high-order visual association cortex and some of its subareas TEa and TEm; TG, architectonic area in the temporal pole; V1-V4, visual areas V1–V4; VIP, ventral intraparietal area; TEO, architectonic area including posterior visual association cortex. The numerals refer to architectonic areas and have the following approximate functional equivalence: 1–3, somatosensory cortex (posterior to the central sulcus); 4, motor cortex; 5, superior parietal lobule; 7a, inferior parietal lobule, visual part; 7b, inferior parietal lobule, somatosensory part; 6, lateral premotor cortex; 8, frontal eye field; 12, part of orbitofrontal cortex; 46, dorsolateral prefrontal cortex.

The organisation of the pathways for food reward in primates including humans shown in Figures 1 and 2 is based on the evidence described next. What is described here refers to primates including humans unless otherwise stated. Largely unimodal taste, olfactory, oral texture and visual sensory inputs that represent what object is represented but not its reward value converge in the orbitofrontal cortex to form multimodal representations that encode food reward. The neuron-level evidence comes from macaques, as this is the best neuron-level evidence that is related to the processing in humans. A unique feature of the approach here is that it combines extensive complementary evidence from the most relevant neuron-level studies with fMRI investigations in humans about food reward systems in the orbitofrontal cortex.

**Fig. 3.**
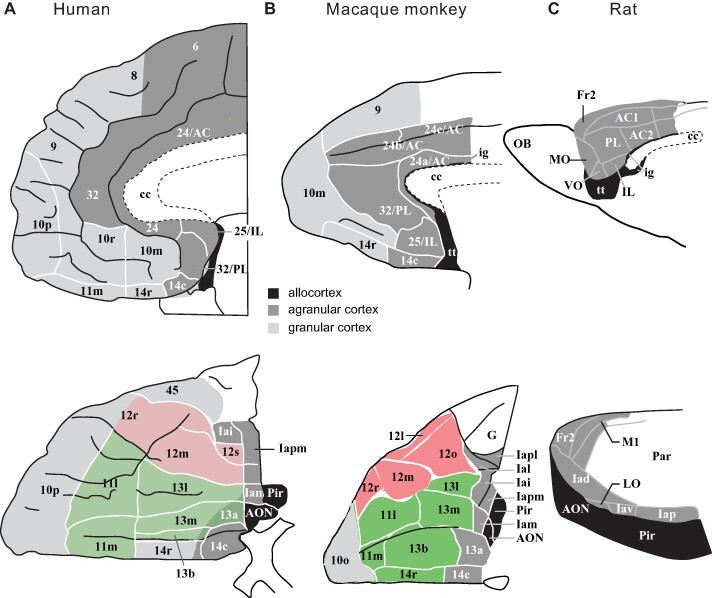
The orbitofrontal (below) and medial prefrontal including anterior cingulate (above) cortical areas in humans, macaque monkeys and rats. (A) Medial (top) and orbital (bottom) areas of the human frontal cortex (Öngür *et al.*, 2003). The medial orbitofrontal cortex is shown in green (areas 13 and 11) and the lateral orbitofrontal cortex in red (area 12). Almost all of the human orbitofrontal cortex except area 13a is granular. Agranular cortex is shown in dark grey. Black shows olfactory regions posterior to the orbitofrontal cortex. The ventromedial prefrontal cortex is the area shown as 10 m and below that towards 11 m. The anterior cingulate cortex comprises areas 32 and 24, with the subgenual area 25. The part of area 45 shown is the orbital part of the inferior frontal gyrus pars triangularis. (B) Medial (top) and orbital (bottom) areas of the macaque frontal cortex. Conventions as in (B). (C) Medial (top) and lateral (bottom) areas of rat frontal cortex [which is thought to have no granular orbitofrontal cortex equivalent to the primate including human granular orbitofrontal cortex areas 11, 13 and 12 (Passingham and Wise, 2012)]. Rostral is to the left in all drawings. Top row: dorsal is up in all drawings. Bottom row: in (A) and (B), lateral is up; in (C), dorsal is up. Not to scale. Abbreviations: AC, anterior cingulate cortex; AON, anterior olfactory nucleus; cc, corpus callosum; Fr2 second frontal area; Ia, agranular insular cortex; ig, induseum griseum; IL, infralimbic cortex; LO, lateral orbital cortex; MO, medial orbital cortex: OB, olfactory bulb; Pr, piriform (olfactory) cortex; PL, prelimbic cortex; tt, tenia tecta; VO, ventral orbital cortex; Subdivisions of areas are labelled caudal (c); inferior (i), lateral (l), medial (m); orbital (o), posterior or polar (p), rostral(r), or by arbitrary designation (a, b). [Adapted from Passingham and Wise (2012)]. (a) Adapted from Ongur, Ferry, and Price (2003) Architectonic subdivision of the human orbital and medial prefrontal cortex, Journal of Comparative Neurology 460: 425–449 (Öngür *et al.*, 2003). (b) Adapted from Carmichael and Price (1994) Architectonic subdivision of the orbital and medial prefrontal cortex in the macaque monkey, Journal of Comparative Neurology 346: 366–402 (Carmichael and Price, 1994). (c) Adapted from Palomero–Gallagher and Zilles (2004) Isocortex, in Paxinos, George ed., The Rat Nervous System, 3e, pp. 729–757 (Palomero-Gallagher and Zilles, 2004).

The orbitofrontal cortex in humans and macaques largely corresponds, as shown in Figure 3. Evidence is described here that the medial orbitofrontal cortex areas 13 and 11 represent food reward value, with convergence of taste, olfactory, visual and somatosensory inputs onto neurons that represent reward value. The medial orbitofrontal cortex represents many other types of reward value (Rolls, 2019a,b; Rolls *et al.*, 2020a; Xie *et al.*, 2021a). The lateral orbitofrontal cortex (red in Figure 3) represents unpleasant stimuli, for example unpleasant odours (Rolls *et al.*, 2003a; Rolls, 2019b), and not obtaining an expected reward such as a food reward (Thorpe *et al.*, 1983) or emotional reward (Kringelbach and Rolls, 2003) or monetary reward (Rolls *et al.*, 2020b; Xie *et al.*, 2021a). The taste, olfactory, visual, somatosensory and auditory anatomical pathways in macaques by which the inputs reach the orbitofrontal cortex are described elsewhere (Ongür and Price, 2000; Rolls, 2015, 2019b, 2021). Tractography (Hsu *et al.*, 2020) and functional connectivity (Du *et al.*, 2020) of the human orbitofrontal cortex show similar connectivity to the macaque. The ventromedial prefrontal cortex (VMPFC) on the medial wall of the frontal lobes (see Figure 3) has connections from the orbitofrontal cortex (Carmichael and Price, 1996; Ongür and Price, 2000; Du *et al.*, 2020; Hsu *et al.*, 2020) and is implicated in decision-making about reward value, rather than representing reward value on a continuous scale as in the orbitofrontal cortex (Rolls and Grabenhorst, 2008; Grabenhorst *et al.*, 2008b; Rolls *et al.*, 2010b,c; Grabenhorst and Rolls, 2011; Glascher *et al.*, 2012; Rolls, 2019b). Rodents may have no granular orbitofrontal cortex areas 13, 11 and 12 corresponding to these areas in primates including humans (see Figure 3), and the whole organisation of the rodent brain systems for taste and related processing is very different to that of macaques, as shown below and elsewhere (Rolls, 2016a,c, 2019b, 2021). Hence, focus on these systems in primates including humans is important for understanding food reward systems in humans, and that is the approach taken here.

## Taste and oral texture food reward in the orbitofrontal cortex

### Taste reward neurons in the orbitofrontal cortex

A secondary cortical taste area in primates was discovered by Rolls and colleagues (Thorpe *et al.*, 1983; Rolls *et al.*, 1989, 1990) in the orbitofrontal cortex (Rolls, 2019b), extending several millimetres in front of the insular primary taste cortex. This is defined as a secondary cortical taste area, for it receives direct inputs from the primary taste cortex, as shown by a combined neurophysiological and anatomical pathway tracing investigation (Baylis *et al.*, 1995). Different neurons in this region respond not only to each of the four classical prototypical tastes sweet, salt, bitter and sour (Rolls *et al.*, 1990, 2003b; Verhagen *et al.*, 2003; Kadohisa *et al.*, 2005b), but also to umami tastants such as glutamate (which is present in many natural foods such as tomatoes, mushrooms and human milk) (Baylis and Rolls, 1991) and inosine monophosphate (which is present in meat and some fish such as tuna) (Rolls *et al.*, 1996a).

In addition, other orbitofrontal cortex neurons respond to water (Rolls *et al.*, 1990), and others to somatosensory stimuli including viscosity, grittiness (Rolls *et al.*, 2003b), astringency as exemplified by tannic acid (Critchley and Rolls, 1996a) and capsaicin (Rolls *et al.*, 2003b; Kadohisa *et al.*, 2004). Fat in food in the mouth is also represented by some neurons in the orbitofrontal cortex (Rolls *et al.*, 1999; Verhagen *et al.*, 2003), and texture is important, for such neurons typically respond not only to foods such as cream and milk containing fat, but also to paraffin oil (which is a pure hydrocarbon) and to silicone oil ((Si(CH_3_)_2_O)_n_). The responses of these oral fat-encoding neurons are not related to free fatty acids such as linoleic or lauric acid (Verhagen *et al.*, 2003; Kadohisa *et al.*, 2005b; Rolls, 2011), and the fat responsiveness of these primate orbitofrontal cortex neurons is therefore not related to fatty acid sensing (Gilbertson *et al.*, 1997; Gilbertson, 1998), but instead to oral texture sensing (Rolls, 2020). The transduction mechanism reflects the coefficient of sliding friction (Rolls *et al.*, 2018), paving the way for the development of new foods with the pleasant mouthfeel of fat but designed nutritional content (Rolls, 2020). In addition, we have shown that some neurons in the orbitofrontal cortex (and also insular taste cortex and amygdala) reflect the temperature of substances in the mouth (Kadohisa *et al.*, 2004, 2005a,b; Verhagen *et al.*, 2004).

**Fig. 4.**
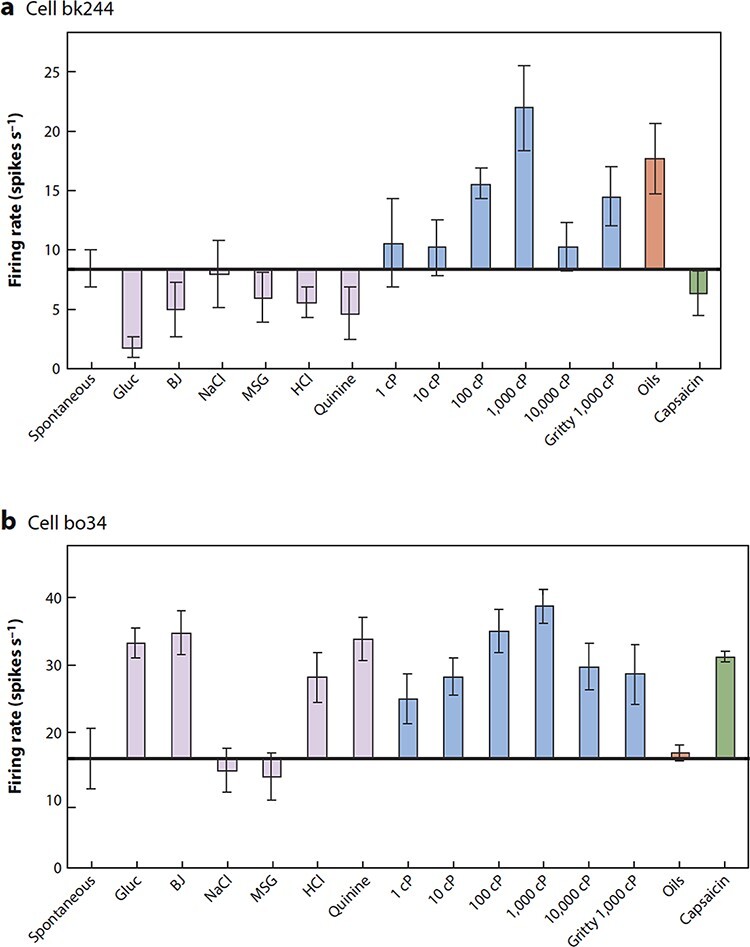
Independent coding of food-related stimuli shown by the responses of two orbitofrontal cortex neurons to taste and oral somatosensory inputs. a. Firing rates (mean ± SEM) of viscosity-sensitive neuron bk244 that did not have taste responses, in that it did not respond differentially to the different taste stimuli. The firing rates are shown for the viscosity series (carboxymethylcellulose 1–10 000 centiPoise), for the gritty stimulus (1000 cP carboxymethylcellulose with Fillite microspheres), for the taste stimuli 1 M glucose (Gluc), 0.1 M NaCl, 0.1 M MSG, 0.01 M HCl and 0.001 M QuinineHCl, and for fruit juice (BJ). Spont = spontaneous firing rate. b. Firing rates (mean ± SEM) of viscosity-sensitive neuron bo34, which had responses to some taste stimuli and had no response to the oils (mineral oil, vegetable oil, safflower oil and coconut oil, which have viscosities that are all close to 50 cP). The neuron did not respond to the gritty stimulus in a way that was unexpected given the viscosity of the stimulus, was taste tuned and did respond to capsaicin. (After Rolls, Verhagen and Kadohisa 2003).

Some of the coding principles are illustrated by the two neurons shown in Figure 4. The two neurons each have their independent tuning to the set of stimuli. It is this independent tuning or coding with sparse distributed representations that underlies the ability of the brain to represent the exact nature of a stimulus or event, and this applies to taste in addition to other sensory modalities including smell (Rolls *et al.*, 1996c, 2010a; Rolls and Treves, 2011; Rolls, 2015, 2016b, 2021). This tuning also provides a foundation for the implementation of sensory-specific satiety (Rolls, 2014, 2015), as described below. Taste responses are found in a large mediolateral extent of the orbitofrontal cortex (Critchley and Rolls, 1996a; Pritchard *et al.*, 2005; Rolls and Grabenhorst, 2008; Rolls, 2008a, 2015).

**Fig. 5.**
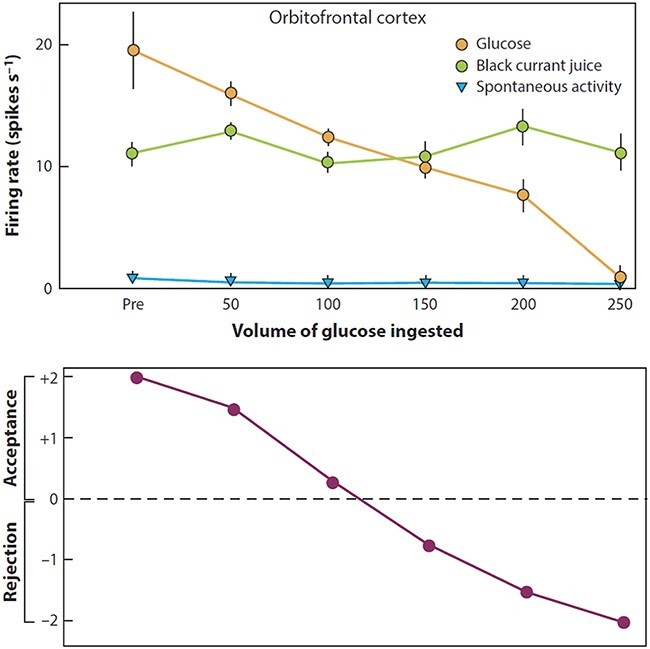
The effect of feeding to satiety with glucose solution on the responses (firing rate ± SEM) of a neuron in the orbitofrontal (secondary taste) cortex to the taste of glucose (open circles) and of blackcurrant juice (BJ). The spontaneous firing rate is also indicated (SA). Below the neuronal response data, the behavioural measure of the acceptance or rejection of the solution on a scale from +2 (strong acceptance) to −2 (strong rejection) is shown. The solution used to feed to satiety was 20% glucose. The monkey was fed 50 ml of the solution at each stage of the experiment as indicated along the abscissa, until he was satiated as shown by whether he accepted or rejected the solution. Pre is the firing rate of the neuron before the satiety experiment started. (After Rolls *et al.*, 1989).

The majority of these orbitofrontal cortex neurons with food-related taste or oral texture responses represent food reward value, in that their responses decrease to zero during feeding to satiety (Critchley and Rolls, 1996c), as illustrated in Figure 5 for the sweet taste of glucose. This procedure is sometimes called reward devaluation and shows that the neurons only respond to food when it is rewarding. Further, feeding to satiety with fat (e.g. cream) decreases the responses of the fat-responsive neurons to zero on the food eaten to satiety, providing evidence that they encode the reward value of fat in the mouth (Rolls *et al.*, 1999).

These taste and oral texture neurons show that sensory-specific satiety is implemented in the orbitofrontal cortex, in that as illustrated in Figure 5, orbitofrontal cortex neurons decrease their responses to the food eaten to satiety, but not to other foods, and this applies to taste neurons and to fat texture neurons (Rolls *et al.*, 1999) (and to neurons that respond to the sight and smell of food, as shown below). In fact this is how Edmund Rolls discovered sensory-specific satiety, one of the most important single factors that influence the amount of food eaten in a meal. The subjective correlate of this modulation is that food tastes pleasant when hungry and tastes hedonically neutral when it has been eaten to satiety. The discovery of sensory-specific satiety was made by recording from neurons in the lateral hypothalamus that receive inputs from the orbitofrontal cortex (Rolls, 1981; Rolls *et al.*, 1986). The hypothalamic neuron being recorded from was responding to the sight of food, a sweet taste, and stopped responding after feeding to satiety with that taste. Rolls at that stage pulled a peanut out of his pocket and offered it to the monkey, and the lateral hypothalamic neuron gave a massive response to the sight of the peanut. It was clear within 3 or 4 presentations that something important was happening here, for the expectation was that after feeding to satiety, hypothalamic reward neurons would no longer respond to food. However, Rolls offered the peanut, and then banana, to the macaque, which avidly ate it. He went on to satiate the monkey with banana and the neuron stopped responding to banana, but still responded to peanuts. And he found that what the hypothalamic neuron still responded to, the monkey would find rewarding and would eat it (Rolls *et al.*, 1986).

Edmund Rolls quickly went on to show with colleagues that sensory-specific satiety was present in humans, and ran generations of Oxford undergraduates on sensory-specific satiety paradigms, showing that they showed sensory-specific satiety for food, and that variety of taste and flavour in a meal was a major factor in influencing how much food is eaten in a meal (Rolls and Rolls, 1977, 1997; Rolls *et al.*, 1981a,b, 1982, 1983a,b, 1984; Hetherington, 2007). Further, it was shown in an Ethiopian refugee camp that there is a long-term form of sensory-specific satiety, which needs to be allowed for when designing foods to be offered on a long time scale (Rolls and De Waal, 1985).

Sensory-specific satiety is present in the primate orbito-frontal cortex, but not at earlier stages of processing including the insular–opercular primary taste cortex (Rolls *et al.*, 1988; Yaxley *et al.*, 1988) and the nucleus of the solitary tract (Yaxley *et al.*, 1985), where the responses reflect factors such as the intensity of the taste, which is little affected by satiety (Rolls *et al.*, 1983c; Rolls and Grabenhorst, 2008). Sensory-specific satiety is probably implemented at least in part by adaptation of the synaptic afferents to orbitofrontal cortex neurons with a time course of the order of the length of a course of a meal (Rolls and Rolls, 1997; Rolls, 2019b). It is complemented by visceral and other satiety-related signals that reach the orbitofrontal cortex (from the nucleus of the solitary tract, via thalamic, insular visceral cortex, and possibly hypothalamic nuclei) and there modulate the representation of food, resulting in an output that reflects the reward (or appetitive) value of each food (Rolls, 2014, 2015, 2016c, 2019b).

Sensory-specific satiety for reward value implemented in the orbitofrontal cortex is found not only for food, but also probably for every other type of reward, and for no punishing stimuli, and is probably a major evolutionary adaptation to help animals to obtain not only a wide range of nutrients, but also the wide range of rewards that are essential for reproductive success (Rolls, 2014, 2019b). Sensory-specific satiety, that is, sensory-specific reward devaluation, is thus a major principle of operation implemented in the orbitofrontal cortex but not at earlier stages of processing in primates (Figure 1).

### Taste neurons before the orbitofrontal cortex

Taste information reaches the orbitofrontal cortex from the insular taste cortex (Baylis *et al.*, 1995). The primary taste cortex is in the anterior (granular) insula and adjoining frontal operculum in macaques (and humans) and receives taste inputs via the nucleus of the solitary tract and the thalamus (VPMpc, ventralposteromedial thalamic nucleus, and pars parvocellularis) (Rolls, 2015). The taste insula contains taste neurons tuned to sweet, salt, bitter, sour (Scott *et al.*, 1986b; Yaxley *et al.*, 1990; Scott and Plata-Salaman, 1999; Rolls and Scott, 2003) and umami as exemplified by monosodium glutamate (MSG; Baylis and Rolls, 1991; Rolls *et al.*, 1996a). It also contains neurons that encode oral somatosensory stimuli including viscosity, fat texture, temperature and capsaicin (Verhagen *et al.*, 2004). Some neurons in the primary taste cortex respond to particular combinations of taste and oral texture stimuli, but macaque insular taste cortex neurons do not respond to olfactory stimuli or visual stimuli such as the sight of food (Verhagen *et al.*, 2004).

Neurons in the primate insular and frontal opercular primary taste cortex do not represent the reward value of taste, that is the appetite for a food, in that their firing is not decreased to zero by feeding the taste to satiety (Rolls *et al.*, 1988; Yaxley *et al.*, 1988). Neural processing peripheral to the primary taste cortex is consistent with this, with taste responses found in the rostral part of the nucleus of the solitary tract (Scott *et al.*, 1986a) that are not influenced by feeding to satiety (Yaxley *et al.*, 1985). This is an important principle of operation of reward systems in primates including humans: sensory processing and perceptual representations take place in cortical areas before the orbitofrontal cortex; and reward processing is implemented in the orbitofrontal cortex (Figure 1). Part of the evolutionary adaptive value of this is that objects can be recognised and their locations, etc. can be remembered even when they are not rewarding, because sensory processing and perception is kept separate from reward value and hedonics in primates including humans, as shown in Figure 1 (Rolls, 2014, 2019b).

### Taste reward activations in humans

fMRI studies in humans are important in that they provide evidence that the same rules of operation of food reward brain systems apply in humans, although they cannot provide anything like the precision of the evidence available from single neuron studies about exactly what is represented in terms of separate stimuli, because tens of thousands of neurons are being averaged across at a time. Human studies are valuable in another way too, for they allow effects of word level cognitive modulations of reward systems to be investigated.

Different regions of the human orbitofrontal cortex can be activated by pleasant (sucrose or glucose) or by aversive (e.g. quinine or sodium chloride) taste stimuli (Zald *et al.*, 1998, 2002; O’Doherty *et al.*, 2001). Umami taste stimuli, of which an exemplar is MSG and which captures what is described as the taste of protein, activate the insular (primary), orbitofrontal (secondary) and anterior cingulate [tertiary (Rolls, 2008a)] taste cortical areas (de Araujo *et al.*, 2003a; Rolls, 2009).

Sensory-specific satiety (and thus reward value) is also reflected in the activations in the human orbitofrontal cortex, in that in a study with real foods with taste, texture and olfactory components, it was found that after feeding to satiety with tomato juice, the activations of the orbitofrontal cortex to tomato juice decreased to zero but not of chocolate milk; whereas after feeding to satiety with chocolate milk, the opposite occurred (Kringelbach *et al.*, 2003). This study thus provided evidence that the subjective pleasantness of the flavour of food and sensory-specific satiety are represented in the human orbitofrontal cortex.

**Fig. 6.**
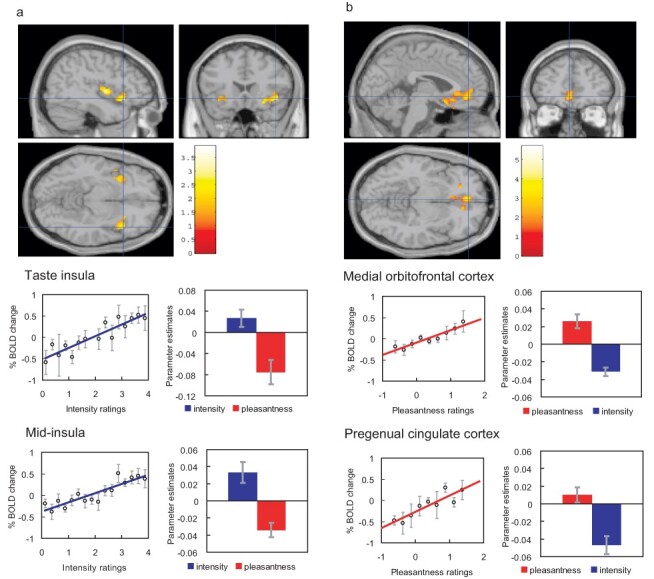
Effect of paying attention to the pleasantness *vs* the intensity of a taste stimulus, MSG. a. Top: A significant difference related to the taste period was found in the taste insula at [42 18–14] *z* = 2.42 *P* < 0.05 (indicated by the cursor) and in the mid insula at [40 −2 4] *z* = 3.03 *P* < 0.025. Middle: Taste Insula. Right: The parameter estimates (mean ± SEM across subjects) for the activation at the specified coordinate for the conditions of paying attention to pleasantness or to intensity. The parameter estimates were significantly different for the taste insula *t* = 4.5, df = 10, *P* = 0.001. Left: The correlation between the intensity ratings and the activation (% BOLD change) at the specified coordinate (*r* = 0.91, df = 14, *P *≪ 0.001). Bottom: Mid Insula. Right: The parameter estimates (mean ± SEM across subjects) for the activation at the specified coordinate for the conditions of paying attention to pleasantness or to intensity. The parameter estimates were significantly different for the mid insula *t* = 5.02, df = 10, *P* = 0.001. Left: The correlation between the intensity ratings and the activation (% BOLD change) at the specified coordinate (*r* = 0.89, df = 15, *P *≪ 0.001). The taste stimulus, MSG, was identical on all trials. b. Top: A significant difference related to the taste period was found in the medial orbitofrontal cortex at [–6 14 −20] *z* = 3.81 *P* < 0.003 (towards the back of the area of activation shown) and in the pregenual cingulate cortex at [–4 46–8] *z* = 2.90 *P* < 0.04 (at the cursor). Middle: Medial orbitofrontal cortex. Right: The parameter estimates (mean ± SEM across subjects) for the activation at the specified coordinate for the conditions of paying attention to pleasantness or to intensity. The parameter estimates were significantly different for the orbitofrontal cortex *t* = 7.27, df = 11, *P* < 10^–4^. Left: The correlation between the pleasantness ratings and the activation (% BOLD change) at the specified coordinate (*r* = 0.94, df = 8, *P *≪ 0.001). Bottom: Pregenual cingulate cortex. Conventions as above. Right: The parameter estimates were significantly different for the pregenual cingulate cortex *t* = 8.70, df = 11, *P *< 10^−5^. Left: The correlation between the pleasantness ratings and the activation (% BOLD change) at the specified coordinate (*r* = 0.89, df = 8, *P* = 0.001). The taste stimulus, 0.1 M MSG, was identical on all trials. [After (Grabenhorst and Rolls, 2008)].

Another type of evidence about reward value in the human orbitofrontal cortex comes from the discovery that the subjective pleasantness of taste stimuli as reported consciously by humans is linearly related to activations in the medial orbitofrontal cortex/ventromedial prefrontal cortex, as shown in Figure 6 (Grabenhorst and Rolls, 2008). The same was found in a region to which the medial orbitofrontal cortex projects (Du *et al.*, 2020; Hsu *et al.*, 2020), the pregenual anterior cingulate cortex (Figure 6) (Grabenhorst and Rolls, 2008), which is involved in actions made to obtain rewarding stimuli (Rolls, 2019c). Consistent with what is found at the neuronal level in primates, activations in the human taste insula were linearly related to the subjective intensity but not pleasantness of the stimulus (Figure 6) (Grabenhorst and Rolls, 2008).

Further evidence about processing in the insular taste cortex is described elsewhere (Small *et al.*, 1999; O’Doherty *et al.*, 2001; de Araujo *et al.*, 2003a, 2012; Grabenhorst and Rolls, 2008; Small, 2010; Rolls, 2015, 2016a,c). In the mid-insular cortex, there is a somatosensory representation of oral texture (de Araujo and Rolls, 2004), which might be unpleasant, and this region can sometimes be activated by taste stimuli as illustrated in Figure 6. If the insular taste cortex in humans is activated by odours, this may be because of taste recalled through backprojection pathways (Rolls, 2016b) from the more anterior agranular insular cortex, which is multimodal (de Araujo *et al.*, 2003b), or from the orbitofrontal cortex. What is encoded in the human insula is the identity/intensity of the taste, not its hedonic/reward value, in that activations in the insula correlate with the intensity ratings but not the pleasantness ratings of the taste (Figure 6) and in that activations in the human insula are modulated by selective attention to the intensity of the taste, as opposed to its pleasantness (Figure 6) (Grabenhorst and Rolls, 2008, 2010; Rolls *et al.*, 2008; Ge *et al.*, 2012; Luo *et al.*, 2013; Rolls, 2013). The texture-related unpleasantness of some oral stimuli is represented in frontal opercular areas that are close to the insular taste cortex (Rolls *et al.*, 2015). This region [and for that matter the taste insula (Verhagen *et al.*, 2004; Kadohisa *et al.*, 2005b)] includes oral somatosensory inputs, and care must be taken to ensure that mouth grimaces, etc. do not occur differentially to the stimuli being used. For example, a small reduction in the activation produced to an aversive taste in this insular/opercular region occurred when it was accompanied by a visual stimulus that led to an expectancy that the taste would not be aversive (Nitschke *et al.*, 2006), but it would be important to show that mouth movements were not the cause of this small effect.

## Olfactory food reward in the orbitofrontal cortex

### Olfactory food reward neurons in the orbitofrontal cortex

Some primate orbitofrontal cortex neurons respond well to olfactory stimuli (Critchley and Rolls, 1996b; Rolls *et al.*, 1996b, 2010a). For many of these olfactory neurons, the response is also related to tastes (Critchley and Rolls, 1996b), and the olfactory representations can be learned by olfactory to taste association learning (Rolls *et al.*, 1996b), providing evidence that the orbitofrontal cortex can remap odours from the olfactory gene–specified representation (Buck and Axel, 1991; Mombaerts, 2006) into a representation where the ‘meaning’ in terms of the association of the odour with other stimuli is paramount. Flavours are built by learning in the orbitofrontal cortex as combinations of taste and olfactory inputs, with oral texture also often being a component (Rolls *et al.*, 1996b). The olfactory to taste association learning is slow, taking 30–60 trials to reverse, so that flavour representations are somewhat stable (Rolls *et al.*, 1996b). The representation of information about odour and taste by primate orbitofrontal cortex neurons (Rolls *et al.*, 1996c, 2010a) is approximately independent by different neurons, in that the information increases approximately linearly with the number of neurons (Rolls *et al.*, 2010a). The Shannon mutual information between the taste and odour stimuli and the neuronal firing is measured in bits (with two bits needed for example to perfectly discriminate four stimuli), and the linear increase in information with the number of neurons (for tens of neurons) provides evidence that the coding by different neurons is independent, enabling the total number of stimuli that can be discriminated to rise exponentially with the number of neurons (because information is a log measure) (Rolls *et al.*, 2010a; Rolls and Treves, 2011; Rolls, 2021). This is a fundamental aspect of brain computation that applies also in the orbitofrontal cortex (Rolls, 2021).

Many primate olfactory orbitofrontal neurons encode the reward value of odour, not only in that their responses often reflect the taste primary reinforcer with which an odour is associated (Critchley and Rolls, 1996b; Rolls *et al.*, 1996b), but also in that their activity is decreased in a sensory-specific satiety way by feeding a particular food to satiety (Critchley and Rolls, 1996c).

### Olfactory food reward activations in the human orbitofrontal cortex

In humans, there is strong and consistent activation of the orbitofrontal cortex by olfactory stimuli (Zatorre *et al.*, 1992; Francis *et al.*, 1999; Rolls *et al.*, 2003a). This region represents the reward value and pleasantness of odour, as shown by a sensory-specific satiety experiment with banana *vs* vanilla odour (O’Doherty *et al.*, 2000), and these reward-specific activations have been confirmed, with evidence too that activations in the pyriform (primary olfactory) cortex were not decreased by odour devaluation by satiety (Gottfried, 2015; Howard *et al.*, 2015). Further, pleasant odours tend to activate the medial, and unpleasant odours the more lateral, orbitofrontal cortex (Rolls *et al.*, 2003a), adding to the evidence that it is a principle that there is a hedonic map in the orbitofrontal cortex, and also in the anterior cingulate cortex, which receives inputs from the orbitofrontal cortex (Rolls and Grabenhorst, 2008; Grabenhorst and Rolls, 2011; Rolls, 2014; Du *et al.*, 2020; Hsu *et al.*, 2020).

The primary olfactory (pyriform) cortex represents the identity and intensity of odour in that activations there correlate with the subjective intensity of the odour, and the orbitofrontal and anterior cingulate cortices represent the reward value of odour, in that activations there correlate with the subjective pleasantness (medially) or unpleasantness (laterally) of odour (Rolls *et al.*, 2003a, 2008, 2009; Grabenhorst *et al.*, 2007; Rolls and Grabenhorst, 2008; Grabenhorst and Rolls, 2011; Rolls, 2014).

## Convergence of olfactory, taste and visual inputs in the orbitofrontal cortex to represent food and its reward value

### Neuronal activity

Taste and olfactory pathways are brought together in the orbitofrontal cortex where flavour is formed by learned associations at the neuronal level between these inputs (see Figure 1) (Rolls and Baylis, 1994; Critchley and Rolls, 1996b; Rolls *et al.*, 1996c). Visual inputs also become associated by learning in the orbitofrontal cortex with the taste of food to represent the sight of food and contribute to flavour (Thorpe *et al.*, 1983; Rolls *et al.*, 1996b). Olfactory-to-taste associative learning by these orbitofrontal cortex neurons may take 30–40 trials to reverse an olfactory-to-taste discrimination task, and this slow learning may help to make a flavour stable (Rolls *et al.*, 1996b). Olfactory neurons are found in a considerable anterior–posterior extent of the primate orbitofrontal cortex, extending far into areas 11 and 14 (Rolls and Baylis, 1994; Critchley and Rolls, 1996b,c; Rolls *et al.*, 1996b,c), and are not restricted to a posterior region as some have thought (Gottfried and Zald, 2005).

Visual-to-taste association learning and its reversal by neurons in the orbitofrontal cortex can take place in as little as one trial (Thorpe *et al.*, 1983; Rolls *et al.*, 1996b; Deco and Rolls, 2005a). This has clear adaptive value in enabling particular foods with a good or bad taste to be learned and recognized quickly, important in foraging and in food selection for ingestion. The visual inputs reach the orbitofrontal cortex from the inferior temporal visual cortex, where neurons respond to visual objects *independently of their reward value (e.g. taste)* as shown by satiety and reversal learning tests (Rolls *et al.*, 1977; Rolls, 2008b, 2012b). The visual-to-taste associations are thus learned in the orbitofrontal cortex (Rolls, 2014, 2019b, 2021). These orbitofrontal cortex visual–taste neurons thus respond to expected value (Rolls, 2014).

### Taste–olfactory convergence shown by activations in humans

Taste and olfactory conjunction analyses, and the measurement of supradditive effects that provide evidence for convergence and interactions in fMRI investigations, showed convergence for taste (sucrose) and odour (strawberry) in the orbitofrontal and anterior cingulate cortex, and activations in these regions were correlated with the pleasantness ratings given by the participants (de Araujo *et al.*, 2003b; Small *et al.*, 2004; Small and Prescott, 2005). These results provide evidence on the neural substrate for the convergence of taste and olfactory stimuli to produce flavour in humans, and where the pleasantness of flavour is represented in the human brain (Rolls, 2014, 2015). The first region where the effects of this olfactory–taste convergence are found is in an agranular part of what cytoarchitecturally is the insula (Ia) that is topologically found in the posterior orbitofrontal cortex, although it is anterior to the insular taste cortex and posterior to the granular orbitofrontal cortex (de Araujo *et al.*, 2003b; Rolls, 2015, 2016a).

McCabe and Rolls (2007) have shown that the convergence of taste and olfactory information in the orbitofrontal cortex appears to be important for the delicious flavour of umami. They showed that when glutamate is given in combination with a consonant, savoury, odour (vegetable), the resulting flavour can be much more pleasant than the glutamate taste or vegetable odour alone, and that this reflected activations in the pregenual cingulate cortex and medial orbitofrontal cortex. The principle is that certain sensory combinations can produce very pleasant food stimuli, which may of course be important in driving food intake, and that these combinations are formed in the brain far beyond the taste or olfactory receptors (Rolls, 2009).

O’Doherty *et al.* (2002) showed that visual stimuli associated with the taste of glucose activate the orbitofrontal cortex and some connected areas, consistent with the primate neurophysiology. Simmons *et al.* (2005) found that showing pictures of foods, compared to pictures of places, can also activate the orbitofrontal cortex. Similarly, the orbitofrontal cortex and connected areas were also found to be activated after presentation of food stimuli to food-deprived subjects (Wang *et al.*, 2004).

## The neuroeconomics of food reward value in the orbitofrontal cortex

The reward value representations in the primate orbitofrontal cortex of taste, olfactory and flavour stimuli are appropriate for economic decision-making in a number of ways (Rolls, 2014, 2015). First, the responses of orbitofrontal cortex neurons reflect the quality of the commodity or ‘good’ (e.g. the sight or taste of food) multiplied by the amount available (Padoa-Schioppa and Assad, 2006; Padoa-Schioppa, 2011; Padoa-Schioppa and Conen, 2017). Moreover, these neurons reflect the value of reward stimuli and not actions made to obtain the rewards (Thorpe *et al.*, 1983; Rolls *et al.*, 1990; Verhagen *et al.*, 2003; Padoa-Schioppa and Assad, 2006; Rolls, 2014, 2019b).

In humans, activations in the ventromedial prefrontal cortex reflect the ‘subjective value’ of foods (where ‘subjective value’ in economics refers to what is chosen by an individual rather than to conscious subjective pleasantness (Rolls, 2014, 2015), measured by the willingness to pay for foods in an auction task (Plassmann *et al.*, 2007)). More generally, there is evidence that the orbitofrontal cortex represents value on a continuous scale, whereas the ventromedial prefrontal cortex is implicated in choices, i.e. decision-making, between stimuli with different values (Rolls and Grabenhorst, 2008; Grabenhorst *et al.*, 2008b, 2010; Grabenhorst and Rolls, 2009, 2011; Rolls *et al.*, 2009, 2010b,c; Rolls *et al.*, 2010d; Glascher *et al.*, 2012; Rolls, 2014, 2019b).

## Representations in the orbitofrontal cortex of reward value on a common scale but not in a common currency

For decision-making, it is important that representations of reward value are on a common scale (so that they can be compared), but are not in a common currency of general reward value, for the specific reward must be represented to guide actions appropriate for obtaining that particular reward (Rolls, 2014, 2015, 2019b, 2021). To investigate whether specific reward representations are on a common scale of reward value, we performed an fMRI study in which we were able to show that even fundamentally different primary rewards, taste in the mouth and warmth on the hand, produced activations in the human orbitofrontal cortex that were scaled to the same range (Grabenhorst *et al.*, 2010). Further fMRI studies are consistent with this (Levy and Glimcher, 2012). These reward value representations in the orbitofrontal cortex are thus in a form suitable for making decisions about whether to for example choose and eat a particular food, with the attractor network decision-making mechanisms now starting to be understood (Wang, 2002; Rolls and Deco, 2010; Rolls *et al.*, 2010b,c,d; Grabenhorst and Rolls, 2011; Rolls, 2014, 2015, 2016b, 2021).

## Top-down cognitive effects on taste, olfactory and flavour food reward processing in the orbitofrontal cortex: a route for social influences on eating

Social factors, for example if a person is informed by another individual or by advertising that a food is in some way good or delicious, can influence eating behaviour. One route by which this can happen is by top-down, cognitive and social, influences on the orbitofrontal cortex food reward system (see Figure 1, ‘Cognitive and attentional top-down bias’). To what extent does cognition influence the hedonics of food-related stimuli, and how far down into the sensory system does the cognitive influence reach? We measured the activation to a standard test odour (isovaleric acid combined with cheddar cheese odour, presented orthonasally using an olfactometer) that was paired with a descriptor word on a screen, which on different trials was ‘Cheddar cheese’ or ‘Body odor’. Participants rated the affective value of the standard test odour, isovaleric acid, as significantly more pleasant when labelled ‘Cheddar Cheese’ than when labelled ‘Body odor’, and these effects reflected activations in the medial orbitofrontal cortex and pregenual cingulate cortex (de Araujo *et al.*, 2005). The implication is that cognitive factors can have profound effects on our responses to the hedonic and sensory properties of food, in that these effects are manifest quite far down into sensory and hedonic processing (in the orbitofrontal cortex, see Figure 1), so that hedonic representations of odours are affected (de Araujo *et al.*, 2005).

**Fig. 7.**
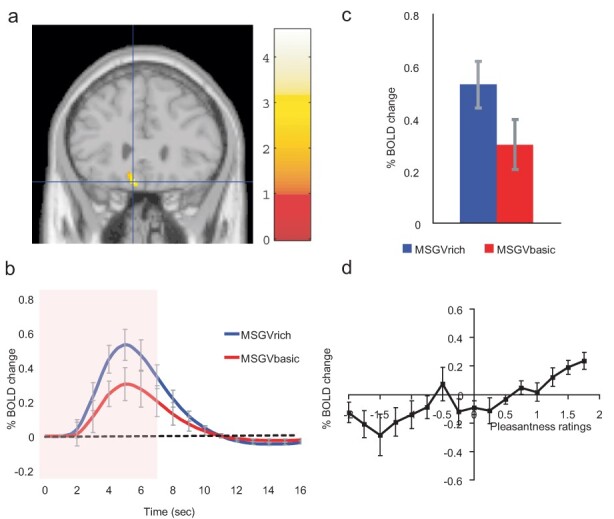
Cognitive modulation of flavour reward processing in the brain. a. The medial orbitofrontal cortex was more strongly activated when a flavour stimulus was labelled ‘rich and delicious flavor’ (MSGVrich) than when it was labelled ‘boiled vegetable water’ (MSGVbasic) [–8 28 −20]. (The flavour stimulus, MSGV, was the taste 0.1 M MSG + 0.005 M inosine 5ʹmonophosphate combined with a consonant 0.4% vegetable odour.) b. The timecourse of the BOLD signals for the two conditions. c. The peak values of the BOLD signal (mean across subjects ± Statistical Parametric Mapping (SEM)) were significantly different (*t* = 3.06, df = 11, *P* = 0.01). d. The BOLD signal in the medial orbitofrontal cortex was correlated with the subjective pleasantness ratings of taste and flavour, as shown by the SPM analysis, and as illustrated (mean across subjects ± SEM, *r* = 0.86, *P* < 0.001). [After (Grabenhorst *et al.*, 2008a)].

Similar cognitive effects and mechanisms have now been found for the taste and flavour of food, where the cognitive word level descriptor was for example ‘rich delicious flavor’ and activations to flavour were increased in the orbitofrontal cortex and regions to which it projects including the pregenual cingulate cortex and ventral striatum, but were not influenced in the insular primary taste cortex where activations reflected the intensity (concentration) of the stimuli (Grabenhorst *et al.*, 2008a) (see Figure 7). Cognitive factors can also influence the release of the hunger-related hormone ghrelin (Crum *et al.*, 2011). If self-control of reward-related processing is required, the dorsolateral prefrontal cortex may be involved in the attentional and related aspects of the processing (Hare *et al.*, 2009; Rolls, 2014; Lowe *et al.*, 2019).

These top-down cognitive word-level effects on food reward systems in the orbitofrontal cortex are likely to be an important route by which social influences, and advertising, can influence food reward value, food choice and the amount of food eaten. Other social influences may well by similar top-down biased competition (Deco and Rolls, 2005b; Rolls, 2013, 2021) modulate the orbitofrontal cortex food reward system in a similar way.

## Top-down selective attention to affective value *vs* intensity biases reward representations in the orbitofrontal cortex: another route for social influences on eating

Selective attention is another way in which social factors may bias the ways in which humans respond to food. When humans are asked to pay selective attention to the pleasantness of a food, there is a top-down modulation of food reward representations in the orbitofrontal cortex to taste, flavour and olfactory food-related stimuli. On the other hand, selective attention to the intensity of the taste, flavour, etc. modulates activations in areas such as the insular primary taste cortex (see Figure 5) (Grabenhorst and Rolls, 2008, 2010; Rolls *et al.*, 2008; Ge *et al.*, 2012; Luo *et al.*, 2013; Rolls, 2013). A source of this top-down modulation by attention of reward processing in the orbitofrontal cortex is the executive system in the dorsolateral prefrontal cortex (Luo *et al.*, 2013), and this is of interest in relation to how the executive system controls behaviour towards rewards (cf. Lowe *et al.*, 2019).

This differential biasing of brain regions engaged in processing a sensory stimulus depending on whether the cognitive demand is for affect-related *vs* more sensory-related processing may be an important aspect of cognition and attention, which have implications for how strongly the reward system is driven by food, and thus for eating and the control of appetite (Grabenhorst and Rolls, 2008, 2011; Rolls *et al.*, 2008; Rolls, 2012a, 2013, 2014). The top-down modulations of processing by cognitive, social and executive function factors have many implications for investigations of taste, olfactory and other sensory processing, for the development of new food products, and for understanding obesity.

## Individual differences in the orbitofrontal cortex food reward system, and their association with obesity and BMI

An important hypothesis is that different humans may have reward systems that differ in how strongly their reward systems are activated, driven by the sensory and cognitive factors that make taste, olfactory and flavour stimuli attractive. In a test of this, we showed that activations to the sight and flavour of chocolate in the orbitofrontal and pregenual cingulate cortex were much higher in chocolate cravers than non-cravers (Rolls and McCabe, 2007), although there were no differences at the level of the insular taste cortex. This provides evidence that differences in specific reward systems, and not necessarily in earlier sensory processing, can lead to individual differences in behaviour to taste, olfactory and flavour stimuli. This is consistent with the hypothesis that part of the way in which evolution results in effective specific reward systems is by utilizing natural variation in these reward systems, and selecting for reward systems that lead to reproductive success (Rolls, 2014, 2018). This concept that individual differences in responsiveness to food reward are reflected in brain activations in regions related to the control food intake (Beaver *et al.*, 2006; Rolls and McCabe, 2007) may provide a way for understanding and helping to control food intake and obesity (Rolls, 2012a, 2014, 2016c).

There is evidence from a number of studies (many relatively small scale with typically fewer than 200 participants) that the structure and function of the orbitofrontal cortex and related regions are related to obesity (Lowe *et al.*, 2019). The following studies are provided as examples. Fibre density measured with tractography was reported to be higher between regions such as the putamen, pallidum and midbrain and the posterior parietal cortex (Gupta *et al.*, 2015). On the other hand, lower grey matter volume of the orbitofrontal cortex, VMPFC, anterior cingulate, striatum and insula is associated with obesity (Shott *et al.*, 2015; Lowe *et al.*, 2019). Higher metabolism of the orbitofrontal cortex (measured with positron emission tomography) was associated with a high BMI in elderly females (Sala *et al.*, 2019). Food addiction scores (*N* = 39) were correlated with greater activation to the anticipation of food of the orbitofrontal cortex, anterior cingulate cortex and amygdala (Gearhardt *et al.*, 2011). Higher responses of the orbitofrontal cortex to visual food cues have been found in obese people (Pursey *et al.*, 2014). Inhibitory control of behaviour by an executive function system in the dorsolateral prefrontal cortex may be one way in which food intake control is maintained (Lowe *et al.*, 2019), and this might include top-down cognitive and executive control of the orbitofrontal cortex.

To investigate whether there are inherent differences between individuals in terms of their orbitofrontal cortex reward systems, we analysed in a very large scale study with 31 536 participants whole-brain functional connectivity in the resting state when no food was available to investigate whether the functional connectivity of parts of the brain is associated with individuals’ liking for sweet foods, and a possible consequence of this, their BMI (Rolls *et al.*, 2021). (Functional connectivity is measured by the correlation between the Blood Oxygenation-Level Dependent (BOLD) signals between each pair of brain areas, with a higher functional connectivity implying that the systems are influencing each other more.) In 31 536 humans from the UK Biobank it was found that increased resting state connectivities of the orbitofrontal cortex/VMPFC especially with the anterior cingulate cortex, were correlated with the liking for sweet foods (False Discovery Rate (FDR) *P* < 0.05). In the same data set, it was found that the functional connectivities of the orbitofrontal cortex were positively correlated with the BMI (FDR *P* < 0.001). Moreover, in a sample of 494 534 people, the ‘liking for sweet foods’ was correlated with their BMI (*r* = 0.06, *P* < 10^−124^) (Rolls *et al.*, 2021).

The correlation between the functional connectivity of the orbitofrontal cortex (relative to that of other brain areas) and the BMI was cross-validated in 569 participants from the Human Connectome Project (Rolls *et al.*, 2021). Further, higher functional connectivity involving the orbitofrontal cortex was associated with high BMI (≥30) compared to a mid-BMI group (22–25). Moreover, relative to other brain areas, low orbitofrontal cortex functional connectivity was associated with low BMI (≤20.8) compared to the mid-BMI group. The latter is interesting, because it is consistent with the hypothesis that lower functional connectivity of the orbitofrontal cortex reward system is associated with low BMI. It was proposed that high BMI relates to increased efficacy of orbitofrontal cortex food reward systems relative to other brain areas, and low BMI to decreased efficacy. It is of interest that this was found in the resting state, when the participants were not being stimulated by the sight or taste of food, so may be an underlying individual difference in brain connectivity (Rolls *et al.*, 2021).

The hypothesis thus is that the increased functional connectivity of the orbitofrontal cortex even when no food is present may be an individual difference that does influence how rewarding food is for an individual, and the increased body weight that may be related to higher eating of such foods. This hypothesis relates to the much broader hypothesis that a driving factor in evolution may be variation in the reward value of different specific types of reward in different individuals, which provides a fundamental basis of personality, that is, individual differences (Rolls, 2014, 2018). In the present case, the implication is that the variation in the connectivity of food reward systems in the brain may lead some individuals to like food more, which of course can be adaptive in some environments, and that this can in some environments, especially when food is highly palatable and readily available, be associated with a high body weight/BMI (Rolls, 2014, 2016c).

Further light is cast on the underlying mechanisms by the finding that it is possible to predict sensation-seeking from the functional connectivity between the medial orbitofrontal cortex and anterior cingulate cortex (Wan *et al.*, 2020). The implication is that the reward-related medial orbitofrontal cortex system by its connections to the action-related cingulate cortex (Rolls, 2019c) can strongly drive reward-related seeking behaviour.

## The orbitofrontal cortex is a food reward system, and not a habit or response or action system

In the primate orbitofrontal cortex, neurons respond to the reward value of sensory stimuli, and do not respond to motor responses (Thorpe *et al.*, 1983; Rolls and Baylis, 1994; Critchley and Rolls, 1996b; Rolls *et al.*, 1996b; Wallis and Miller, 2003; Padoa-Schioppa and Assad, 2006; Grattan and Glimcher, 2014). Reward value is a property of stimuli, and this is what is represented in the primate including human orbitofrontal cortex (Rolls, 2019b, 2021).

One way in which reward systems influence behaviour is via the cingulate cortex, which implements goal-related learning of actions that is under the control of the reward value of the goal, for example obtaining food (Rolls, 2021) (see Figure 1). The concept is that the posterior cingulate cortex receives information about actions being performed from the parietal cortex; receives information about whether the action was rewarded from the orbitofrontal cortex; learns the appropriate actions to obtain the rewards and avoid the punishers; and sends the output from the midcingulate cortex to premotor cortical areas (Rolls, 2019c, 2021).

A second way in which the orbitofrontal cortex influences behaviour is via its projections to the striatum, to reinforce stimulus–response habits, which once stamped in, result in the responses being performed when the stimulus is received even if the stimulus is no longer rewarding (Rolls, 2014, 2021). The reinforcement signal from the orbitofrontal cortex may act directly in the striatum, but also via its influence on dopamine neurons via the ventral striatum and habenula (Rolls, 2017, 2021). It is normally the case that motivated behaviour is performed for the reward or goal, and it is only when a habit or stimulus–response behaviour becomes established that eating is no longer under the control of the reward (Berridge *et al.*, 2009); so normally goal-directed ‘liking’ predicts motivation or ‘wanting’, but when the habit system is involved, the behaviour can become unlinked from liking (Rolls, 2014, 2015).

As described below, the rodent orbitofrontal cortex is not functionally homologous to the primate orbitofrontal cortex, because the rodent orbitofrontal cortex has representations of behavioural responses (Wilson *et al.*, 2014; Sharpe *et al.*, 2015; Rolls, 2019b, 2021).

## Orbitofrontal cortex food reward systems and their relation to conditioned appetite and conditioned satiety

Gut and other post-ingestive consequences on a longer time scale can influence food reward mechanisms. For example, if the food has a high energy value, then gradually humans learn to eat less of that flavour of food, in what is termed conditioned satiety (Booth, 1985). If the food has a low energy value, then more of it is consumed by learning over a few meals, and this is termed conditioned appetite (or ‘appetition’) (Booth, 1985; Sclafani, 2013). Thus, post-ingestive consequences of eating can by learning influence the sensory (taste, olfactory, etc.) reward value of food, and the same type of associative learning between the flavour of a food and its post-ingestive consequences can account for the findings (de Araujo *et al.*, 2020) that hungry animals learn from gut signals to choose a food with significant energy content (they ‘like and want it’). Thus, associative learning between the flavour of a food and its post-ingestive consequences appears to be the mechanism (Sclafani, 2013), rather than gut signals being what is primarily rewarding (de Araujo *et al.*, 2020). Consistent with my view, food reward that can reinforce actions is not found when the food directly enters the stomach unless large volumes are delivered (Nicolaidis and Rowland, 1976, 1977; Rolls, 2014), partly because the time course is too slow for each aliquot that enters the stomach to act as a discrete reward for an action. So even if vagal afferent stimulation can induce reward (Han *et al.*, 2018), the time course of this route and the fact that food accumulates in the stomach and drains steadily into the duodenum makes this a poor system for reinforcing individual actions, but instead a suitable slow signal for slow associative learning of associations between flavour reward in the mouth and food in the gut. Consistent with this evidence, humans report that intragastric feeding is neither pleasant nor rewarding, as is well known in clinical medicine (Rolls, 2014). In more detail, during sham feeding when food drains from the stomach, whether the individual eats is under the control of the sight, smell and taste of the food, which acts as the reward for eating. A tiny drop of food is sufficient to reward and maintain the behaviour. If the food is no longer delivered to be tasted and swallowed, then the sham feeding soon stops. That is the evidence that it is the sight, smell and taste of food that provide food reward (Rolls, 2014). Moreover, the subjective pleasantness of the food is related to its flavour as signalled by taste, oral texture and odour. By contrast, when food is delivered directly into the stomach, it is not very rewarding, in that enormous quantities, for example one-quarter of the capacity of the stomach, have to be delivered in order for the animal to slowly learn to deliver food to the stomach (Nicolaidis and Rowland, 1976, 1977; Rolls, 2014).

By contrast, food in the gut acts as a satiety signal, to switch off reward. A very telling observation is that if after eating to satiety the stomach is drained of food, feeding resumes immediately (Gibbs *et al.*, 1981). This proves that a gut signal acts by producing satiety and by influencing the operationally defined reward value of food, which is whether an individual works for the taste, smell and sight of the food, i.e. for the sensory properties of the food. There is much evidence that modulation of the sensory reward or appetitive value of a food by gut signals is also relevant to clinical conditions, including obesity (Monteiro and Batterham, 2017; Makaronidis and Batterham, 2018).

Strong further evidence for the importance of taste, olfactory, visual and oral texture cues in producing food reward value comes from studies of sensory-specific satiety and the effects of variety on food intake (Rolls *et al.*, 1981a,b, 1983c, 1989; Critchley and Rolls, 1996c; Rolls and Rolls, 1997; Kringelbach *et al.*, 2003), which cannot be accounted for by the gut reward signals that have been discussed (De Araujo *et al.*, 2020). Rolls’ theory, therefore, is that taste, olfactory, oral texture and visual food reward systems determine whether food is eaten, and that gut signals modulate these sensory food reward systems, both by short-term satiety signals and by longer-term conditioning of the reward value of the sensory properties (taste, texture, smell and sight) of particular foods (Rolls, 2014, 2016c). That is, while humans are eating in a meal, the reward value and pleasure of food are produced by its sensory properties including its taste, texture, smell and sight. This reward value is reduced by sensory-specific satiety [implemented it is suggested by the adaptation of synapses bringing these sensory inputs onto neurons in the primate orbitofrontal cortex (Rolls, 2014)], by gut signals including gastric distension which rely on food entering the duodenum (Gibbs *et al.*, 1981) and by post-absorptive effects that accumulate during a meal (Rolls, 2014). Over the longer term, the reward value of the sensory properties of a food can be conditioned by its nutritional consequences (Booth, 1985; Sclafani, 2013; Rolls, 2014, 2016c), and that is a relatively slow conditioning effect on the reward value produced by the sight, taste, texture and smell of food.

The evidence thus is that the taste and flavour (including its oral texture) of a food is a primary, unlearned reward, and that the reward value can be modulated later in life by associative learning between the taste and flavour of food and its post-ingestive consequences. Further evidence for an innate liking for different tastes, which shows that taste is a primary, unlearned, reinforcer, is that very young rat pups display different reactivities to different tastes for at least some of which there has been no opportunity for conditioning (Kehoe and Blass, 1985). Further consistent evidence from humans is that we found greater reactivity of the agranular insular taste area, and the supracallosal cingulate cortex where aversive stimuli are represented (Rolls, 2019c), to the taste and texture of vegetable juice in young adults of student age than in older age groups (Rolls *et al.*, 2015). This probably relates to the well-known dislike in young individuals of vegetables such as Brussels sprouts that are somewhat bitter (Rolls *et al.*, 2015). [The agranular insular taste area is just anterior to the primary insular taste cortex in which the unpleasantness of these stimuli was not represented (Rolls *et al.*, 2015), consistent with the evidence about the insular taste cortex representing taste identity and intensity but not hedonics described above.] The implications are that stimuli such as taste and oral texture are primary reinforcers and that later in life post-ingestive gut-related consequences of the food eaten can be associated by learning with the taste of food that has been recently eaten in the processes known as conditioned appetite and conditioned satiety (Sclafani, 2013; Rolls, 2014).

## Unpleasant stimuli and non-reward in the lateral orbitofrontal cortex

Many unpleasant stimuli, including unpleasant odours, are represented in the lateral orbitofrontal cortex area 12 (Rolls *et al.*, 2003a, 2020a; Grabenhorst and Rolls, 2011; Rolls, 2019b), which then connects with the supracallosal anterior cingulate cortex (Rolls, 2019c; Du *et al.*, 2020; Hsu *et al.*, 2020). This part of the orbitofrontal cortex via its influence of the supracallosal anterior cingulate cortex may contribute to food choice by representing unpleasant aspects of food stimuli, such as in young adults the bitterness present in vegetable juice, to which older participants are much less sensitive (Rolls *et al.*, 2015).

Different neurons in the orbitofrontal cortex respond when a visually signalled expected taste reward is not obtained, that is, to negative reward prediction error (Thorpe *et al.*, 1983; Rolls and Grabenhorst, 2008; Rolls, 2014, 2019b). Activations in the lateral orbitofrontal cortex occur when an expected reward is not obtained, and reversal of choice should occur (Kringelbach and Rolls, 2003; Rolls *et al.*, 2020b). Moreover, damage to the human orbitofrontal cortex impairs this reward reversal behaviour and also is associated with impulsiveness, which may reflect insensitivity to non-reward (Rolls *et al.*, 1994; Berlin *et al.*, 2004, 2005; Hornak *et al.*, 2004). This system may be involved in controlling food choice behaviour, by stopping behaviour when eating may be appropriate. Indeed, frontotemporal dementia is associated with disorders of eating of this type (Ahmed *et al.*, 2019) that may be accounted for in the way just described. Similarly, undersensitivity or poor top-down control of this lateral orbitofrontal cortex system may contribute to disinhibited over-eating and obesity. Over-sensitivity and over-connectivity of this lateral orbitofrontal cortex system non-reward system are associated with depression (Rolls, 2018; Rolls *et al.*, 2020a; Xie *et al.*, 2021b).

## Food reward systems in humans and other primates compared to those in rodents

Emphasis is placed here on research in primates and humans, because there is evidence that the rodent taste and food reward systems operate somewhat differently (Rolls, 2014; Rolls, 2015, 2016a, 2021). In brief, the taste system is different in rodents in that there is a pontine taste area, which then projects subcortically, but in primates there is no pontine taste area and cortical processing is performed first (Scott and Small, 2009; Small and Scott, 2009; Rolls, 2016a). Second, in rodents, the taste and olfactory systems are modulated peripherally [in the nucleus of the solitary tract and the olfactory bulb, respectively (Pager *et al.*, 1972; Palouzier-Paulignan *et al.*, 2012)] by hunger so that reward is represented peripherally and is entangled with sensory processing, whereas in primates and humans food perception is separated from its reward value (Figure 1) (Rolls, 2014). A perceptual correlate of this is that when humans feed to satiety, the intensity of the flavour changes very little, whereas the pleasantness of the flavour decreases to zero (Rolls *et al.*, 1983c; Rolls and Rolls, 1997), showing that in humans’ perceptual representations of taste and olfaction are kept separate from hedonic representations. This is adaptive, in that we do not go blind to the sight, taste and smell of food after eating it to satiety and can, therefore, still learn about where food is located in the environment even when we are not hungry (Rolls, 2014). Third, the orbitofrontal cortex is very little developed in rodents (with only an agranular part) (Wise, 2008; Passingham and Wise, 2012) (Figure 3), yet is one of the major brain areas involved in taste and olfactory processing, and emotion and motivation, in primates including humans (Rolls, 2014, 2019b, 2021). Fourth, the rodent visual system is far less developed than the primate visual system (Rolls, 2021), and the reward value of the sight of food is very important in finding and selecting food in humans and other primates and is a major influence on the primate orbitofrontal cortex reward system, as described above. These findings make the rodent taste, olfactory and visual systems a poor model of neural food reward processing in humans, and for that reason emphasis is placed here on discoveries in primates and humans (Rolls, 2014; Rolls, 2015, 2016a, 2019b, 2021).

## The amygdala

The amygdala is a structure in the temporal lobe with somewhat similar connections to the orbitofrontal cortex (see Figure 1). The amygdala has been present in evolution for much longer than the primate orbitofrontal cortex and appears to differ from the orbitofrontal cortex in that it cannot implement one-trial, rule-based, visual discrimination reversal when the taste or flavour associated with the visual stimulus is reversed (Rolls, 2014, 2021). The primate amygdala contains neurons that respond to taste and oral texture (Sanghera *et al.*, 1979; Scott *et al.*, 1993; Kadohisa *et al.*, 2005a,b). Some neurons respond to visual stimuli associated with reinforcers such as taste, but do not reflect the reinforcing properties very specifically, do not rapidly learn and reverse visual-to-taste associations, and are much less affected by reward devaluation by feeding to satiety than are orbitofrontal cortex neurons (Sanghera *et al.*, 1979; Yan and Scott, 1996; Wilson and Rolls, 2005; Kadohisa *et al.*, 2005a, 2005b; Rolls, 2014). The primate orbitofrontal cortex appears to be much more closely involved in flexible (rapidly learned, and affected by reward devaluation) reward representations than is the primate amygdala (Rolls, 2014, 2019b, 2021).

Fat texture, oral viscosity and temperature, for some neurons in combination with taste, and also the sight and smell of food, are represented in the macaque amygdala (Rolls and Scott, 2003; Kadohisa *et al.*, 2005a,b). Interestingly, the responses of these amygdala neurons do not correlate well with the preferences of the macaques for the oral stimuli (Kadohisa *et al.*, 2005b), and feeding to satiety does not produce the large reduction in the responses of amygdala neurons to food (Yan and Scott, 1996; Rolls and Scott, 2003) that is typical of orbitofrontal cortex neurons.

 We found activation of the human amygdala by the taste of glucose (Francis *et al.*, 1999). Extending this study, O’Doherty *et al.* (2001) showed that the human amygdala was as much activated by the affectively pleasant taste of glucose as by the affectively negative taste of NaCl, and thus provided evidence that the human amygdala is not especially involved in processing aversive as compared to rewarding stimuli. Zald et al. (1998; 2002) also showed that the human amygdala responds to aversive (e.g. quinine) and to sucrose taste stimuli.

Rolls has compared and contrasted the roles of the orbitofrontal cortex *vs* the amygdala in behaviour (Rolls, 2014, 2019b, 2021).

## Beyond reward value to decision-making in the ventromedial prefrontal cortex

Representations of the reward value of food, and their subjective correlate the pleasantness of food, are fundamental in determining appetite and processes such as food-related economic decision-making (Padoa-Schioppa, 2011; Padoa-Schioppa and Cai, 2011; Rolls, 2014). But after the reward evaluation, a decision has to be made about whether to seek for and consume the reward. We are now starting to understand how the brain takes decisions (Wang, 2002; Rolls and Deco, 2010; Deco *et al.*, 2013; Rolls, 2014, 2021), and this has implications for whether a reward of a particular value will be selected (Rolls and Grabenhorst, 2008; Rolls, 2008b, 2014, 2021; Rolls and Deco, 2010; Grabenhorst and Rolls, 2011; Deco *et al.*, 2013).

A tier of processing beyond the orbitofrontal cortex, in the ventromedial prefrontal cortex area 10 (see Figure 3), becomes engaged when choices are made between odour stimuli based on their pleasantness (Grabenhorst *et al.*, 2008b; Rolls *et al.,* 2010b,c,d) (Tier 3 in Figure 1). For example, activations in this area are larger when humans make a decision about which of two odours they prefer, compared to only rating the odours on a continuous scale of reward value (Grabenhorst *et al.*, 2008b). The activations found during this decision-making are similar to those predicted from the attractor network model of decision-making (Rolls *et al.*, 2010b,c; Rolls, 2021).

## Conclusions

Analysis of the orbitofrontal cortex shows how it represents the reward value of the taste, texture, smell and sight of food. This is a key system involved in the control of food intake. Moreover, individual differences in the orbitofrontal cortex reward system are correlated with the liking for sweet foods and BMI (Rolls *et al.*, 2021), indicating that this food reward system plays a role in the control of body weight. Although the correlation was not high in this investigation, it was highly significant, and was based on the reported liking for sweet foods, which is only one simple and limited measure of food reward, and higher correlations might be expected with fuller measures of food reward.

The analysis of food reward systems in the orbitofrontal cortex leads to better understanding of the factors that are likely to influence eating behaviour, including sensory-specific satiety and variety in what is readily available; the high palatability of many modern foods in relation to satiety signals that evolved before these highly palatable foods became available; and social, cognitive and executive control influences on orbitofrontal cortex food reward systems. In fact, it has been suggested that in order to control obesity, it may be important to understand all the factors that may contribute to high food intake, because unless all are controlled, overeating may occur (Rolls, 2016c). The factors are described in more detail elsewhere (Rolls, 2016c), but include genetic factors; endocrine factors and how they affect brain reward systems as well as metabolism; the delicate balance between orbitofrontal cortex food reward systems that may be overdriven in the modern environment, and satiety signals; the high palatability of modern foods; sensory-specific satiety and the effect of variety on food intake; food saliency and portion size, effects that relate to the importance of the sight of food in humans and that relate to advertising; the fact that food is readily available at many times of the day, which may disturb the normal timing between meals; the high energy density of foods that make it difficult for satiety signals to operate before energy intake is high; a high eating rate, which can have similar effects; and stress (Rolls, 2016c).
